# Functional Identification and Structural Analysis of a New Lipoate Protein Ligase in *Mycoplasma hyopneumoniae*

**DOI:** 10.3389/fcimb.2020.00156

**Published:** 2020-04-21

**Authors:** Kemeng Zhu, Huan Chen, Jin Jin, Ning Wang, Guixing Ma, Jiandong Huang, Youjun Feng, Jiuqing Xin, Hongmin Zhang, Henggui Liu

**Affiliations:** ^1^State Key Laboratory of Veterinary Biotechnology, Harbin Veterinary Research Institute, The Chinese Academy of Agricultural Sciences, Harbin, China; ^2^Department of Biology, Guangdong Provincial Key Laboratory of Cell Microenvironment and Disease Research, Shenzhen Key Laboratory of Cell Microenvironment and SUSTech-HKU Joint Laboratories for Matrix Biology and Diseases, Southern University of Science and Technology, Shenzhen, China; ^3^School of Biomedical Sciences, Li Ka Shing Faculty of Medicine, The University of Hong Kong, Hong Kong, China; ^4^Institute of Synthetic Biology, Shenzhen Institutes of Advanced Technology, Chinese Academy of Sciences, Shenzhen, China; ^5^Department of Pathogen Biology and Microbiology, Zhejiang University School of Medicine, Hangzhou, China

**Keywords:** *Mycoplasma hyopneumoniae*, lipoate protein ligase, lipoate metabolism, crystal structure, *M. hyopneumoniae* GcvH

## Abstract

*Mycoplasma hyopneumoniae* (*M. hyopneumoniae*) is the causative agent of pandemic pneumonia among pigs, namely, swine enzootic pneumonia. Although *M. hyopneumoniae* was first identified in 1965, little is known regarding its metabolic pathways, which might play a pivotal role during disease pathogenesis. Lipoate is an essential cofactor for enzymes important for central metabolism. However, the lipoate metabolism pathway in *M. hyopneumoniae* is definitely unclear. Here, we identified a novel gene, lpl, encoding a lipoate protein ligase in the genome of *M. hyopneumoniae* (Mhp-Lpl). This gene contains 1,032 base pairs and encodes a protein of 343 amino acids, which is between 7.5 and 36.09% identical to lipoate protein ligases (Lpls) of other species. Similar to its homologs in other species, Mhp-Lpl catalyzes the ATP-dependent activation of lipoate to lipoyl-AMP and the transfer of the activated lipoyl onto the lipoyl domains of *M. hyopneumoniae* GcvH (Mhp H) *in vitro*. Enzymatic and mutagenesis analysis indicate that residue K56 within the SKT sequence of Mhp H protein is the lipoyl moiety acceptor site. The three-dimensional structure showed typical lipoate protein ligase folding, with a large N-terminal domain and a small C-terminal domain. The large N-terminal domain is responsible for the full enzymatic activity of Mhp-Lpl. The identification and characterization of Mhp-Lpl will be beneficial to our understanding of *M. hyopneumoniae* metabolism.

**Summary:**

Lipoic acid is an essential cofactor for the activation of some enzyme complexes involved in key metabolic processes. Lipoate protein ligases (Lpls) are responsible for the metabolism of lipoic acid. To date, little is known regarding the Lpls in *M. hyopneumoniae*. In this study, we identified a lipoate protein ligase of *M. hyopneumoniae*. We further analyzed the function, overall structure and ligand-binding site of this protein. The lipoate acceptor site on *M. hyopneumoniae* GcvH was also identified. Together, these findings reveal that Lpl exists in *M. hyopneumoniae* and will provide a basis for further exploration of the pathway of lipoic acid metabolism in *M. hyopneumoniae*.

## Introduction

*Mycoplasma hyopneumoniae* (*M. hyopneumoniae*) is the causative agent of a worldwide and chronic pneumonia in pigs known as swine enzootic pneumonia (Mare and Switzer, 1965; Maes et al., 2008). *M. hyopneumoniae* infection is associated with economic losses due to reduced daily weight gain and feed efficiency, increased mortality, and production costs because of medication and vaccination. Additionally, pigs are predisposed to infection with viruses and other bacteria after infection by *M. hyopneumoniae*. Previous studies have shown that *M. hyopneumoniae* is very difficult to isolate from the infected lungs of pigs and its growth is slow. These phenomena indicate that the metabolism of *M. hyopneumoniae* has specific characteristics. However, little is known about the metabolism of *M. hyopneumoniae*.

Lipoic acid is a sulfur-containing cofactor that is universally required for aerobic metabolism (Smith et al., 2004). Lipoic acid is required for the activity of enzymes involved in oxidative and single carbon metabolism, such as dehydrogenase (PDH), 2-oxoglutarate dehydrogenase (2-OGDH), and branched-chain keto-acid dehydrogenase (BCKDH). Specific enzymes catalyze the covalent attachment of lipoic acid to the acyltransferase (E2) subunit or H protein of the glycine cleavage system (GcvH) via an amide linkage between the ε-amino group of a specific lysine residue of these proteins and the lipoic acid carboxyl group (Reed and Hackert, 1990; Fujiwara et al., 1992; Reche and Perham, 1999). The metabolism of lipoic acid has been clearly elucidated in *Escherichia coli (E. coli)*. When lipoic acid is available in the environment, *E. coli* LplA catalyzes both the activation of lipoate to lipoyl-AMP and the subsequent transfer of the activated lipoyl moiety to an acceptor protein with lipoyl domains (LDs) (Reed et al., 1958; Morris et al., 1994, 1995). If exogenous lipoic acid is absent, *E. coli* LipB and LipA will initiate the lipoate synthesis pathway *in vivo* (Cronan et al., 2005). In this synthesis pathway, LipB functions as an octanoyl-acyl carrier protein (ACP) transferase that transfers the octanoyl moiety from the fatty acid biosynthetic intermediate octanoyl-ACP to the LD of a lipoate acceptor protein (Morris et al., 1995; Zhao et al., 2005). LipA then catalyzes the insertion of a sulfur into octanoylated domains to yield dihydrolipoyl-LD, which is further oxidized to lipoyl-LD (Douglas et al., 2006). In addition to *E. coli*, the enzymes involved in lipoate modification of proteins have been found in most other organisms, including *Streptomyces coelicolor* (Cao and Cronan, 2015), *Mycobacterium tuberculosis* (Ma et al., 2006), *Bacillus subtilis* (Christensen et al., 2011b), *Listeria monocytogenes* (Christensen et al., 2011a), *Thermoplasma acidophilum* (Kim et al., 2005), *Chlamydia trachomatis serovar* L2 (Ramaswamy and Maurelli, 2010), *Plasmodium falciparum* (Gunther et al., 2007), *Saccharomyces cerevisiae* (Hermes and Cronan, 2013), plants (Ewald et al., 2014), bovines (Fujiwara et al., 1997), and humans (Cao et al., 2018b).

*M. hyopneumoniae* is a prokaryotic organism. Although *M. hyopneumoniae* was discovered as early as 1965 (Mare and Switzer, 1965), the enzymes responsible for the lipoate modification of proteins are unclear. In this study, we explore important enzymes that participate in the metabolism of lipoic acid in *M. hyopneumoniae*. Here, a putative lipoate protein ligase (Lpl) was found in the genome of *M. hyopneumoniae* by sequence analysis. This putative protein was expressed and purified. Functional analysis confirmed that the protein exerts a function similar to that of Lpl *in vitro*. We then solved the crystal structure of the protein. The structure indicated that this putative Lpl has a three-dimensional structure similar to that of the *E. coli* LplA, although their protein sequences share minimal identity. As Lpl is an important enzyme in lipoic acid metabolism, these results will facilitate our understanding of lipoic acid metabolism in *M. hyopneumoniae*.

## Materials and Methods

### Plasmid Construction

All plasmids used and constructed in this study are shown in Table 1. In brief, the putative *Mhp-lpl* (MHP_RS01680) and *Mhp H* gene, in which the TGA stop codons in the ORF were replaced with TGG, were commercially synthesized after being optimized with E. coli codon. The synthesized *Mhp-lpl* was amplified with the primer pairs P1-F/P1-R and inserted into pET32a(+) between NdeI and XhoI sites to obtain the recombinant plasmid pX1. The synthesized *Mhp H* was amplified with the primer pairs P2-F/P2-R and inserted into pET32a(+) between NdeI and XhoI sites to obtain the recombinant plasmid pX2. The genes of *E. coli* LplA and GcvH were amplified from the DH5α strain with the primer pairs P3-F/P3-R and P4-F/P4-R, respectively. Both genes were inserted into pET32a(+) between NdeI and XhoI sites to obtain the recombinant plasmids pX3 and pX4, respectively. To express the large (1-254 aa) and small domains (260-344 aa) of Mhp-Lpl, the two domains were amplified from the synthesized *Mhp-Lpl* with the designed primer pairs P1-F/P5-R and P6-F/P1-R and inserted into pET32a(+) between NdeI and XhoI sites to obtain the recombinant plasmids pX5 and pX6, respectively. All primer sequences used in this research are listed in Table 2.

**Table 1:** Plasmids used in this research.

pET32a	T7 promoter expression vector	Lab stock
pX1	pET32a encoding Mhp*-Lpl*	This study
pX2	pET32a encoding MhpH	This study
pX3	pET32a encoding *E. coli* LplA	This study
pX4	pET32a encoding *E. coli* GcvH	This study
pX5	pET32a encoding Mhp-lpl large domain	This study
pX6	pET32a encoding Mhp-lpl small domain	This study

**Table 2:** Primers used in this research.

**Primer name**	**Primer sequence(5^′^ → 3^′^)**
P1-F	TTTCATATGTACCTGATTGAACCGAAAC
P1-R	TTTCTCGAGCAGCAGCAGGTTACAAATTT
P2-F	TTTCATATGAAGAAGATCGCAAAT
P2-R	TTTCTCGAGAAAATCTTCCAGCTCATCAAAT
P3-F	TTTCATATGTCCACATTACGCCTGCT
P3-R	TTTCTCGAGCCTTACAGCCCCCGCCA
P4-F	TTTCATATGAGCAACGTACCAGCAGAA
P4-R	TTTCTCGAGCTCGTCTTCTAACAATGCTTCGTA
P5-R	TTTCTCGAGGCTCAGGCCCCACACAAAAT
P6-F	TTTCATATGAATTATAGTTTTAATCGCAGT

*The underlined nucleotide sequences are the endonuclease sites*.

### Protein Expression and Purification

To express and purify the recombinant proteins with a hexahistidine-containing tag at the C terminus, the recombinant vectors were transformed into *E. coli* BL21 (DE3) cells and cultured in Luria broth at 37°C. When the cells reached 0.5 at OD_600_, a final concentration of 1 mM isopropyl 1-thio-β-D-galactopyranoside (IPTG) was added. After incubating for an additional 6 h at 37°C, the cells were harvested and lysed by sonication in lysis buffer (20 mM Tris-HCl, pH 7.5, 500 mMNaCl) containing 20 mM imidazole. The crude lysate was centrifuged at 12,000 g for 20 min. The supernatant was applied to an affinity chromatography column of Ni-NTA-agarose. The proteins were eluted with lysis buffer containing 500 mM imidazole. The protein used for crystallization was further purified by HiTrap SP column chromatography (GE Healthcare) and size-exclusion column HiLoad™ 16/600 Superdex™ 200 pg chromatography (GE Healthcare). This protein was concentrated to 180 mg/ml and stored at −80°C for later use.

### Production of a Monoclonal Antibody in Mice

To produce specific monoclonal antibodies against Mhp-Lpl in mice, 6- to 7-week-old mice were immunized with the purified protein emulsified with complete or incomplete Freund's adjuvant. After three immunizations with an interval of 2 weeks, the mice were sacrificed to isolate spleen cells. The hybridomas were produced and screened according to the protocol described previously (Chen et al., 2018). In brief, splenocytes and mouse SP2/0 myeloma cells were fused at a ratio of 5:1 using PEG 1450 at 37°C. The resulting hybridoma cells were plated onto ten 96-well plates and initially grown in RPMI 1640 medium supplemented with 10% fetal bovine serum for 24 h. Then, hybridoma cells were selected with hypoxanthine-aminopterin-thymidine (HAT)-conditioned medium for 2 weeks. The supernatant from each well was assayed by ELISA plated with Mhp-Lpl. The positive hybridoma cells were harvested and plated onto 96-well plates at a density of 0.5 cells/well. The supernatant from the plated hybridomas was assayed again as described above. The positive hybridoma was expanded, cultured and frozen in liquid nitrogen.

### Assay of Enzymatic Function of Lpl *in vitro*

The activity of the Lpl or the separate domains of Lpl was analyzed as described previously (Afanador et al., 2014; Cao and Cronan, 2015). In brief, the purified Lpl or the separate domains of Lpl were incubated in reaction buffer (50 mM sodium phosphate, pH 7.0) containing 5 mM ATP, 5 mM DTT, 1 mM MgCl_2_, 1 mM lipoic acidand 20 μM apo-GcvH. After incubation at 37°C for 4 h, the lipoylation of apo-GcvH protein in the reaction was determined with rabbit anti-lipoic acid polyclonal antibodies (Abcam) using a western blot assay.

### Western Blot Analysis

To determine the lipoylation of apo-GcvH protein or expression of Lpl in *M. hyopneumoniae*, a western blot assay was carried out as described previously (Cao and Cronan, 2015). In brief, proteins were loaded and separated on a 12% SDS-polyacrylamide gel and transferred by electrophoresis to nitrocellulose membranes (Millipore) for 25 min at 25 V. The membranes were blocked with 5% non-fat milk in PBST buffer (137 mM NaCl, 2.7 mM KCl, 10 mM Na_2_HPO_4_, 2 mM KH_2_PO_4_, 0.01% Tween-20). The membranes were first stained with a rabbit anti-lipoic acid primary antibody (1:7500; Abcam) or mouse anti-Lpl monoclonal antibodies for 30 min. After washing three times with PBST, Odyssey Dylight 800-conjugated goatanti-rabbit or mouse IgG antibody (1:8000; Abcam) was added and incubated for 30 min. After washing three times, the membranes were analyzed by using Odyssey CLX Image Studio software.

### Mutagenesis of Mhp H Protein

Sixteen lysine residues (K2, K3, K13, K36, K40, K41, K45, K51, K56, K62, K66, K78, K81, K90, K95, and K97) in the Mhp H protein were individually mutated to alanine residues. Each mutant gene was commercially synthesized and inserted into pET32a between the first NdeI and XhoIrestriction sites. The mutant Mhp H proteins were named K2A, K3A, K13A, K36A, K40A, K41A, K45A, K51A, K56A, K62A, K66A, K78A, K81A, K90A, K95A, and K97A.

### Crystallization, Diffraction Data Collection and Structure Determination

Crystals of Mhp-Lpl were obtained by the hanging drop vapor diffusion method. In brief, protein solutions at 60 mg/ml were mixed in a 1:1 ratio with reservoir solutions (0.2M (NH_4_)_2_SO_4_, 0.1 M sodium acetate, pH 4.0, 20% PEG 2000 MME) and incubated at 16 °C. Crystals appeared in 1 day and grew to a suitable size for data collection in 1–3 days. Native crystals were harvested and soaked in cryoprotectant (0.2M (NH_4_)_2_SO_4_, 0.1M sodium acetate, pH 4.0, 20% PEG 2000 MME and 16% glycerol) and then quickly immersed in liquid nitrogen. For the assistance of phasing, the dipicolinate lanthanide complex Nd(DPA)_3_ (Pompidor et al., 2010) at a final concentration of 125 mM was added to the cryoprotectant, and the crystals were soaked for 30 min before freezing. X-ray diffraction data were collected at 100 K on the BL17U1 and BL19U1 beamlines at the Shanghai Synchrotron Radiation Facility (SSRF) (Wang et al., 2018) and processed using HKL2000. The structure was solved using the SAS protocol of Auto-Rickshaw: the EMBL-Hamburg automated crystal structure determination platform (Panjikar et al., 2005).

The input diffraction data were prepared and converted for use in Auto-Rickshaw using programs of the CCP4 suite (Collaborative Computational Project, 1994). FA values were calculated using the program SHELXC (Sheldrick, 2010). Based on an initial analysis of the data, the maximum resolution for substructure determination and initial phase calculation was set to 2.8 Å. All 15 heavy atoms requested were found using the program SHELXD (Schneider and Sheldrick, 2002). The correct hand for the substructure was determined using the programs ABS (Hao, 2004) and SHELXE (Sheldrick, 2002). Initial phases were calculated after density modification using the program SHELXE (Sheldrick, 2002). The two-fold non-crystallographic symmetry (NCS) operator was found using the program RESOLVE (Terwilliger, 2003). Density modification, phase extension and NCS-averaging were performed using the program DM (Cowtan and Zhang, 1999). A total of 30.90% of the model was built using the program ARP/wARP (Perrakis et al., 2001; Morris et al., 2003). The initial model was further built and refined with Phenix (Adams et al., 2010). The data collected from native crystals were used for final refinement using the model from Nd-derivative data. After alternative cycles of refinement with Refmac5 (Murshudov et al., 2011) and manual building in Coot (Emsley et al., 2010), clear positive electron density was identified at the putative active site of Mhp-Lpl, and lipoyl-AMP was modeled into the density. The final model was evaluated by MolProbity, showing that 96% of the residues were in the Ramachandran favored region and allowed region. Data collection and model refinement statistics are listed in Table 3. The coordinates and structure factors were deposited in the Protein Data Bank with the access code 6JOM.

**Table 3:** Data collection and refinement statistics.

	**Mhp-Lpl native**	**Mhp-Lpl SAD**
**Data collection**		
Space group	P4_1_2_1_2	P4_1_2_1_2
Cell dimensions		
*a, b, c* (Å)	100.32, 100.32, 155.58	102.44, 102.44, 161.47
α, β, γ (°)	90.00, 90.00, 90.00	90.00, 90.00, 90.00
Resolution (Å)	50~2.45 (2.54~2.45)^*^	50~2.30 (2.38~2.30)
*R*_merge_	0.060 (0.758)	0.104 (0.729)
*I* /σ*I*	40.4 (3.5)	29.1 (5.5)
Completeness (%)	99.6 (99.1)	97.8 (95.2)
Redundancy	14.9 (14.9)	18.2 (18.7)
**Refinement**		
Resolution (Å)	50~2.45	50~2.30
No. reflections	29572 (2730)	36085
*R*_work_ / *R*_free_	0.2007/0.2683	0.1959/0.2631
No. atoms	5736	
Protein	5431	
LAQ	68	
Water	237	
Ramachandran favored (%)	95.87	
allowed (%)	3.98	
outliers (%)	0.15	
*B*-factors	67.10	
Protein	67.41	
LAQ	73.75	
Water	59.93	
**R.m.s. deviations**		
Bond lengths (Å)	0.010	
Bond angles (°)	1.75	

* *Values in parentheses are for highest-resolution shell*.

## Results

### Sequence Analysis of a Putative Lpl From *M. hyopneumoniae*

**Figure 1:**
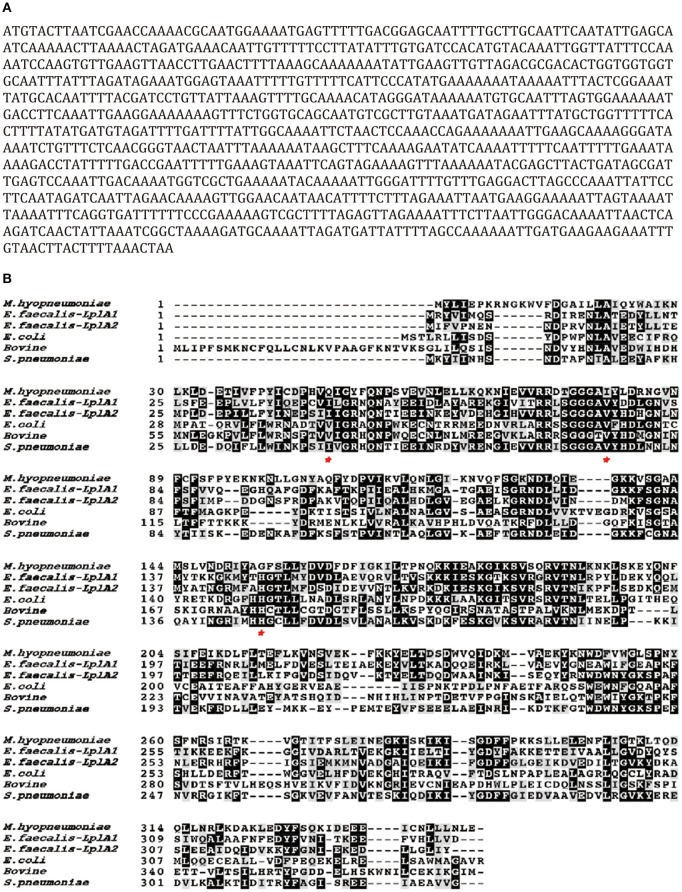
Sequence analysis of a putative Lpl (MHP_RS01680) from *M. hyopneumoniae*.**(A)** The open reading frame of the putative Lpl cloned from the genome of *M. hyopneumoniae*. **(B)** Protein sequence alignment among the putative Lpl from *M. hyopneumoniae* and LplA molecules from *Enterococcus faecalis (E. faecium), Streptococcus pneumoniae* (*S. pneumoniae), Escherichia coli* (*E.coli*) and *Bostaurus* (bovine). Residues labeled with an asterisk implicate key differences at ligand binding sites between Mhp*-*Lpl and LplA molecules from other species.

**Figure 2:**
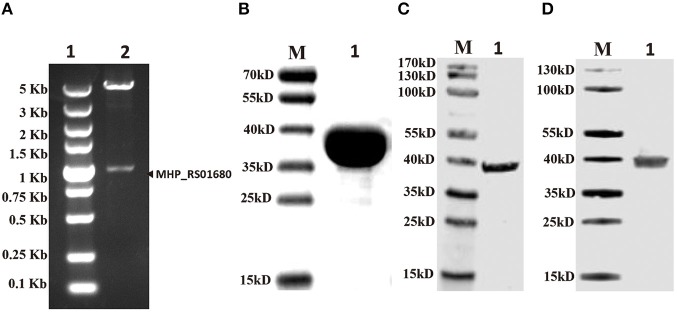
Confirming putative Lpl (MHP_RS01680) existence in *M. hyopneumoniae*. **(A)** The gene fragment of MHP_RS01680 from *M. hyopneumoniae* was inserted into the prokaryotic expression vector pET32a between NdeI and XhoI sites. The constructed recombinant plasmid was identified by double digestion with NdeI and XhoI enzymes. Lane 1, DL5000 DNA marker. Lane 2, digested recombinant plasmid. **(B)** MHP_RS01680-encoded protein was expressed in the *E. coli* BL21 (DE) strain, purified and analyzed by SDS-PAGE. Lane M, protein marker (kD); lane 1, the purified protein. **(C)** The MHP_RS01680-encoded protein was probed with the produced monoclonal antibody in the western blot assay. Lane M: protein marker (kD); lane 1, the purified MHP_RS01680-encoded protein. **(D)** The MHP_RS01680-encoded protein in *M. hyopneumoniae* was analyzed by western blotting using the specific monoclonal antibody. Lane M, protein marker (kD); lane 1, The MHP_RS01680-encoded protein in *M. hyopneumoniae*.

Lpl is an important enzyme for the metabolization of lipoic acid, which plays a role as a cofactor in eukaryotes, most bacteria and some archaea. To address the Lpl in *M. hyopneumoniae*, the genome of *M. hyopneumoniae* strain 232 (accession number: NC_006360) was searched. MHP_RS01680, which was annotated as a lipoate protein ligase, was found. The nucleotide sequence of MHP_RS01680 contains 1032 bp encoding 343 amino acids (Figure 1A). The amino sequence shares 7.5% to 36.09% identity with LplA sequences of other species deposited in GenBank. The protein sequence alignment showed that the sequences with higher identity include LplA2 (36.09%) and LplA1 (35.58%) from *Enterococcus faecalis*, LplA from *Streptococcus pneumoniae* (33.54%), *E. coli* (26.49%), and *Bos Taurus* (24.17%), respectively (Figure 1B). There are 11 *M. hyopneumoniae* strains of which the genomes have been deposited into GeneBank. Protein alignment of Lpls among these strains indicated that they are highly conserved with sequence identity between 89 and 99.9% (Figure S1). As the gene was derived from automated computational analysis using a gene prediction method, it is not known whether this gene is expressed *in vivo*. To test the expression of the putative protein in *M. hyopneumoniae*, the MHP_RS01680 nucleotide sequence with all TGA codons within the open reading frame (ORF) substituted with TGG was synthesized and inserted into the pET32a vector. The protein encoded by MHP_RS01680 was induced and expressed in the *E. coli* BL21 strain and subsequently purified (Figures 2A,B). The purified protein was used to immunize mice to produce a monoclonal antibody using a hybridoma technique. As shown in Figure 2C, the produced monoclonal antibodies could specifically recognize the purified proteins in the western blot assay. This result indicated that the monoclonal antibody was successfully produced. The monoclonal antibody was further used to detect MHP_RS01680 expression in *M. hyopneumoniae*. As shown in Figure 2D, when the total proteins of the *M. hyopneumoniae* J strain were extracted and subjected to western blotting, a protein band with a size of ~40 kD appeared, which is equal to the size deduced from the MHP_RS01680 nucleotide sequence. This result confirmed the expression of MHP_RS01680 in *M. hyopneumoniae*.

### Functional Analysis of the Putative *M. hyopneumoniae* Lpl *in vitro*

**Figure 3:**
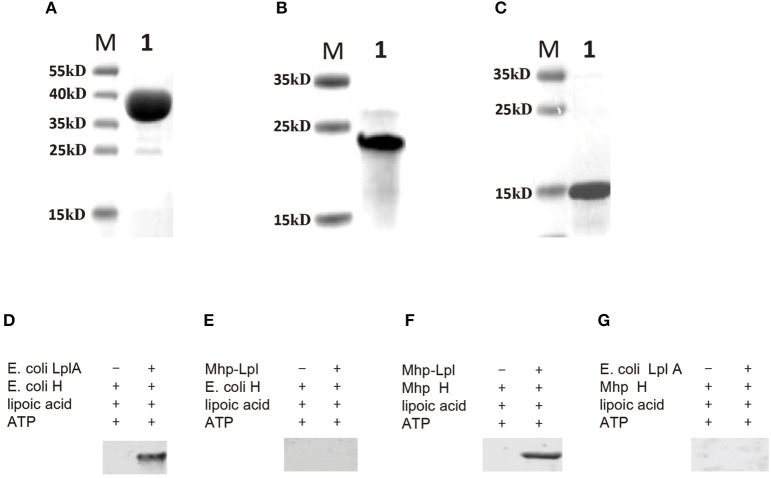
Functional analysis of Lpl from *M. hyopneumoniae*. **(A)** The *E. coli* LplA gene was inserted into the prokaryotic expression vector and induced expression in the *E. coli* BL21(DE3) strain. The expressed protein was purified and analyzed with SDS-PAGE assay (Lane 1), Lane M is protein marker (kD). **(B)** The *E. coli* H gene was inserted into the prokaryotic expression vector and induced expression in the *E. coli* BL21(DE3) strain. The expressed protein was purified and analyzed with SDS-PAGE assay (Lane 1), Lane M is protein marker (kD). **(C)** The *Mhp H* gene was inserted into the prokaryotic expression vector and induced expression in the *E. coli* BL21(DE3) strain. The expressed protein was purified and analyzed with SDS-PAGE assay (Lane 1), Lane M is protein marker (kD). **(D)** Analysis of the transfer of lipoic acid to *E. coli* GcvH (*E. coli* H) protein by *E. coli* LplA. **(E)** Analysis of lipoic acid transferred to *E. coli* H protein by Mhp-Lpl. **(F)** Analysis of the transfer of lipoic acid to the Mhp H protein by Mhp-Lpl. **(G)** Analysis of lipoic acid transferred to Mhp H protein by *E. coli* LplA.

Although MHP_RS01680 is annotated as a lipoate protein ligase in *M. hyopneumoniae*, it is not known whether this protein is able to transfer lipoic acid to the acceptor protein, which is the functional phenotype of Lpl proteins. To characterize the function of MHP_RS01680, *E. coli* GcvH protein and LplA were expressed and purified (Figures 3A,B). The lipoic acid transfer assay was carried out *in vitro*. As shown in Figures 3D,E, although *E. coli* LplA could successfully transfer lipoic acid to *E. coli* GcvH, the MHP_RS01680-encoded protein failed. As *E. coli* GcvH is able to accept the lipoate transferred by Lpl from other species (Cao and Cronan, 2015), this phenomenon probably resulted from two possibilities: the MHP_RS01680-encoded protein does not function as an Lpl or this protein cannot recognize *E. coli* GcvH as a lipoate acceptor protein. To further investigate these questions, the *M. hyopneumoniae gcvh* genes in the genomes of different strainswere analyzed. It was found that *M. hyopneumoniae* GcvH (Mhp H) proteins are highly conserved (Figure S2). According to this result, Mhp H from one strain was expressed and purified (Figure 3C). The lipoate transfer assay was carried out. As shown in Figure 3F, lipoic acid was successfully transferred to the Mhp H protein by the MHP_RS01680-encoded protein. This result indicates that the MHP_RS01680-encoded protein is an Lpl in *M. hyopneumoniae* (Mhp-Lpl). However, *E. coli* LplA fail to catalyze Mhp H modification by lipoic acid (Figure 3G).

### Identification of the Lipoate Acceptor Site on GcvH Recognized by Mhp-Lpl

**Figure 4:**
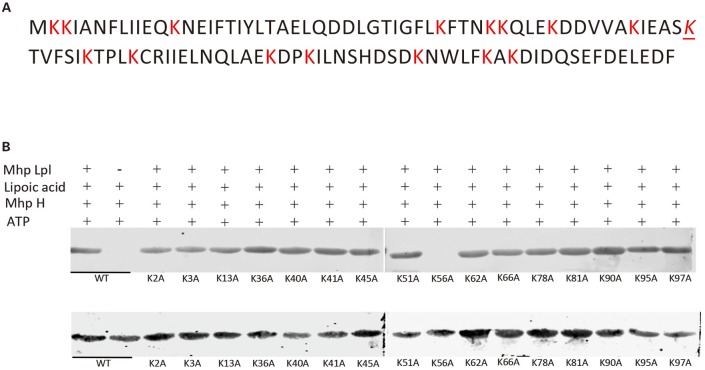
Identification of the acceptor site of lipoic acid on the Mhp H protein. **(A)** The sequence of the Mhp H protein. All lysine (K) residues are marked with red color. The italicized and underlined K is the lysine residue accepting the lipoyl moiety. **(B)** The lysines on the Mhp H protein were individually mutated into alanine residues. All mutated and wild type (wt) Mhp H proteins were expressed and purified. Their ability to accept the lipoyl moiety was analyzed *in vitro*. The mutated Mhp H proteins in upper and lower images were probed with anti-lipoic acid and anti-His tag antibodies, respectively, in the western blot assay.

In the glycine cleavage system, GcvH, as an acceptor protein for lipoate, is generally conserved. *E. coli* GcvH accepts the lipoate transferred by Lpl proteins from other species (Cao and Cronan, 2015). Here, Mhp-Lpl cannot transfer lipoate to *E. coli* GcvH, indicating that the recognition site on the acceptor protein for Mhp-Lpl is different from that of the Lpls from other species. As the lipoate is transferred to the lysine residue on the acceptor protein, we first screened the lysine residues accepting the transferred lipoyl moiety on the Mhp H protein. A total of 16 lysine residues on the Mhp H protein were individually mutated to alanine residues. All mutant Mhp H proteins were used to analyze the ability of accepting the lipoyl moiety. As shown in Figure 4, of all the mutant Mhp H proteins, only the mutant protein K56A was not able to accept the lipoyl moiety. This result indicated that K56 was the amino residue accepting the lipoyl moiety transferred by Mhp-Lpl. This residue is within the SKT amino acid sequence (Figure 4A).

### Overall Structure of Mhp-Lpl

**Figure 5:**
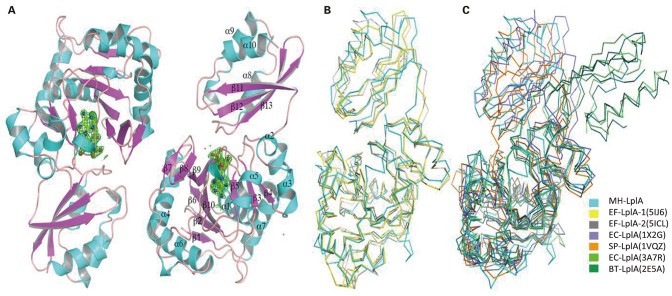
Overall structure of Mhp-Lpl and structural superposition with LplA molecules from other species. **(A)** Overall structure of Mhp-Lpl. Mhp-Lpl is shown as a cartoon with helices and β-sheets colored in cyan and magenta, respectively. Electron densities corresponding to bound ligands are shown as green mesh at 3.0 σ. Structural superposition of Mhp-Lpl with LplA molecules from *E. faecalis* (Ef-LplA, PDB codes 5IJ6 and 5ICL) **(B)** and *E. coli* (Ec-LplA, 1X2G and 3A7R), *S. pneumoniae* (Sp-LplA, 1VQZ) and bovine (Bt-LplA, 2E5A) **(C)**. All molecules are shown as Cα tracing for clarity and colored as indicated.

Mhp-Lpl was crystallized in the space group of P4_1_2_1_2, and there are two Mhp-Lpl molecules in the asymmetric unit (Figure 5A). Although the amino acid sequence identity between Mhp-Lpl and LplA-2 from *E. faecalis* (Ef-LplA-2) is 36.09%, the structure determination by molecular replacement method using Ef-LplA-2 as a searching model was unsuccessful, indicating possible structural variation between these two proteins. The lanthanide compound Nd(DPA)_3_ was soaked into the Mhp-Lpl crystals, and the structure was solved by a single-wavelength anomalous dispersion method. Most of the amino acid residues could be clearly traced, except residues between 173 and 183, part of the adenylate binding loop following the helix α5, which is also disordered in Ef-LplA-2 (PDB code 5IBY) and LplA from *E. coli* (Ec-LplA, PDB code 1X2H).

Mhp-Lpl is composed of a large N-terminal domain (residues 1–254), a small C-terminal domain (residues 260–344), and a short polypeptide (residues 255–259) between the two domains (Figure 5A). The N-terminal domain comprises two β-sheets, a large mixed β-sheet comprising sevenstrands (β1, β2, β6-β10) and a small mixed β-sheet comprising three strands (β3–β5), with seven α-helices (α1–α7) surrounding the β-sheets. The C-terminal domain comprises three helices (α8–α10) and a β-sheet composed of three strands (β11–β13). The two molecules in the asymmetric unit are almost identical, with a root mean square deviation (rmsd) value of 0.42 for all superimposable Cα atoms. The overall structure of Mhp-Lpl is similar to those of EF-LplA-1 in complex with lipoate (PDB code 5IJ6) and EF-LplA-2 in complex with lipoyl-AMP (PDB 5ICL), with rmsd values of 1.48 and 1.73 for approximately 300 superimposable Cα atoms, respectively (Figure 5B). In contrast, Mhp-Lpl shows large variations with LplA molecules from *E. coli* (Ec-LplA, PDB code 1X2G, unliganded form) (Fujiwara et al., 2005) and *S. pneumoniae* (Sp-LplA, PDB code 1VQZ, unliganded form) with rmsd values of 2.88 and 2.61, respectively (Figure 5C). The relatively large rmsd values also indicate why the molecular replacement method failed to yield a reasonable solution. Although Mhp-Lpl binds to lipoyl-AMP at its active site (described in the following section), it adopts a closed conformation similar to those of Ef-LplA-2 (PDB code 5ICL) and the unliganded Ec-LplA (PDB code 1X2G). Upon ligand binding, Ec-LplA (PDB code 3A7R) (Fujiwara et al., 2010) undergoes dramatic conformational changes and adopts a stretched conformation similar to that of bovine LplA (BT-LplA, PDB code 2E5A) (Figure 5C) (Fujiwara et al., 2007), whereas both unliganded and liganded bovine LplA adopt a stretched conformation and no dramatic conformational changes are present upon ligand binding (Fujiwara et al., 2010).

### Ligand Binding Site of Mhp-Lpl

**Figure 6:**
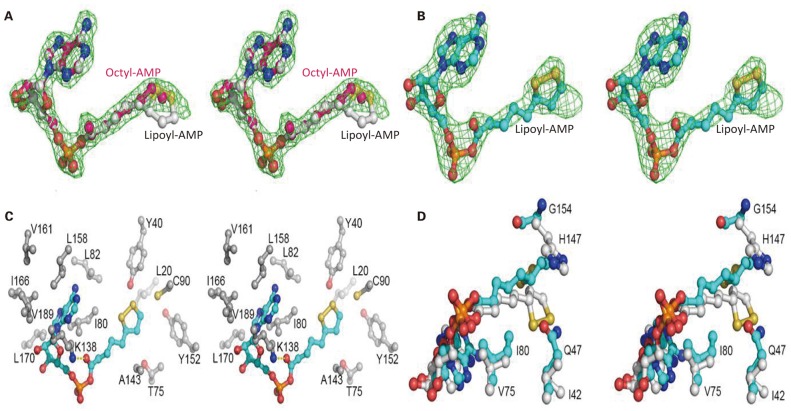
Structural analysis of the active site of Mhp-Lpl. The Fo-Fc map is shown as green mesh at 3.0 σ in **(A,B)**. Lipoyl-AMP, octyl-AMP and residues at the active site are shown as ball-and-stick models. **(A)** Electron densities for intrinsically bound AMP-fatty acids. The electron densities reveal a linear fatty acid conjugated with AMP. Lipoyl-AMP from Ec-LplA (PDB code 5ICL) and octyl-AMP from Ec-LPlA (PDB code 3A7A) are modeled into the density for comparison. **(B)** Electron densities for lipoyl-AMP binding at the active site of Mhp-Lpl. Mhp-Lpl reacted with ATP and lipoate and then crystallized. Lipoyl-AMP has been modeled into the density. **(C)** Interaction of lipoyl-AMP with surrounding residues at the active site of Mhp-Lpl. Only hydrophobic interactions are shown for clarity. The Nε of residue K138 also forms hydrogen bonds with the carbonyl oxygen of lipoate. **(D)** Comparison of lipoyl-AMP molecules at the active sites of Mhp-Lpl and Ef-LplA-2 (PDB code 5ICL). Lipoyl-AMP and residues in Mhp-Lpl and Ef-LplA-2 are shown with cyan and gray carbon atoms, respectively.

**Figure 7:**
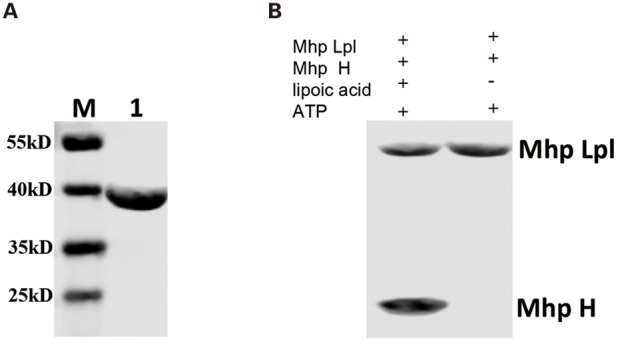
Functional analysis of AMP-fatty acid as a substrate. **(A)** Mhp-Lpl expressed in *E. coli* was probed with anti-lipoic acid polyclonal antibodies in the western blot assay. Lane M is protein marker (kD). Lane 1 is the purified Mhp-Lpl from the BL21(DE3) strain. **(B)** Mhp-Lpl was expressed and purified from *E. coli*. The transfer of fatty acid binding at the active site of Mhp-Lpl was analyzed in the presence or absence of lipoic acid.

Mhp-Lpl was expressed in *E. coli* and purified by Ni-NTA affinity chromatography, cation exchange column sulfopropyl (SP) chromatography and size-exclusion column chromatography. The structural determination clearly showed an extra positive density at the putative active site (Figure 5A) between the two β-sheets in the N-terminal domain resembling AMP conjugated with a fatty acid. The AMP part could be fitted into the density unambiguously, while the fatty acid part indicated a linear structure in contrast to the ring structure in lipoic acid (Figure 6A). This fatty acid is longer than octoic acid (Figure 6A), and the separation and identification of the bound molecule by mass spectrometry was unsuccessful. As an anti-lipoic acid monoclonal antibody can also recognize this molecule (Figure 7A), we sought to determine whether this molecule could be transferred to the Mhp H protein as a substrate. As shown in Figure 7B, although the molecule existed at the active site in the purified Mhp-Lpl protein expressed in *E. coli*, the Mhp H protein could not be modified in the absence of lipoic acid. This result indicates that this bound molecule is not the functional substrate for Mhp-Lpl.

To clearly see the interaction between Mhp-Lpl and lipoic acid, Mhp-Lpl was first reacted with ATP and lipoic acid and then crystallized after further purification. As shown in Figure 6B, lipoyl-AMP could be modeled into the density, despite its partial occupancy. Similar to lipoyl-AMP molecules in Ec-LplA (PDB code 3A7R) and Ef-LplA-2 (PDB code 5ICL), the lipoyl-AMP in Mhp-Lpl also adopts a U-shaped conformation at the bifurcated active site sandwiched between the lipoate-binding loop (residues 72-83, RRDTGGGAIYLD) and the large β-sheet of the N-terminal domain. The AMP part of lipoyl-AMP forms extensive hydrophobic interactions with the surrounding residues, including I80, L82, K138, L158, I166, L170, and V189 (Figure 6C). Among these residues, K138 forms an additional hydrogen bond with the carbonyl oxygen of lipoyl-AMP and plays essential roles in both lipoate adenylation and lipoate transfer reactions as shown in *E. coli* LplA (Fujiwara et al., 2010). In addition to the nitrogen atoms in the adenine ring, the ribose and phosphate groups also form hydrogen bonds with neighboring residues or water molecules. The aliphatic moiety of lipoic acid is enclosed in a hydrophobic tunnel formed by residues L20, Y40, T75, C90, A143, and Y152 (Figure 6C).

Structural superposition revealed several key differences at the ligand binding sites between Mhp-Lpl and LplA molecules from other species. The residues corresponding to residue Q47 in Mhp-Lpl all have a small side chain, such as isoleucine or valine, in the LplA proteins from other species (Figure 1B). The large-sized side chain of residue Q47 in Mhp-Lpl pushes the dithiolane ring of the lipoyl moiety away and flips toward the large β-sheet in the N-terminal domain (Figure 6D). Correspondingly, the otherwise conserved histidine residue in LplA from other species at position 154 (Figure 1B) is replaced by a glycine residue in Mhp-Lpl, which enables the encapsulation of the dithiolane ring of the lipoyl moiety. Moreover, the conserved valine residue in the lipoate-binding loop (77 in Ec-LplA and 75 in Ef-LplA-2, Figure 1B) is substituted with an isoleucine residue (I80, Figures 6C,D), which enhances the hydrophobic interaction with lipoyl-AMP and explains why it is difficult to remove the unidentified AMP-fatty acid conjugate from Mhp-Lpl expressed in *E. coli*.

### The Large Domain Is Responsible for the Overall Lipoylation of Mhp H

**Figure 8:**
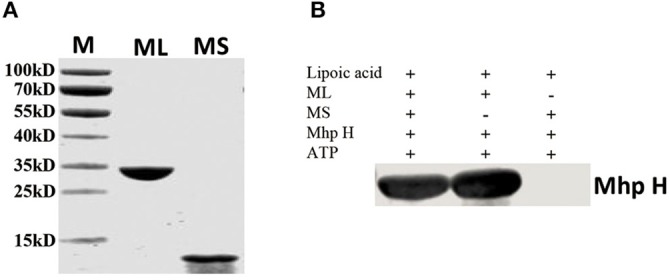
Functional analysis of the large and small domains of Mhp-Lpl. **(A)** SDS-PAGE analysis of purified large and small domains of Mhp-Lpl expressed in *E. coli*. M is protein marker (kD). ML is the large domain of Mhp-Lpl. MS is the small domain of Mhp-Lpl. **(B)** Lipoate modification of the Mhp H protein by a large or small domain or both was submitted to western blotting. The lipoyl moiety conjugated with Mhp H protein was probed with anti-lipoic acid polyclonal antibodies.

The overall lipoylation reaction of GcvH contains two steps: activation of lipoic acid with ATP to form lipoyl-AMP and the lipoyl moiety transfer to GcvH protein. Similar to other Lpls, Mhp-Lpl molecule contains two domains, the large and small domains. To examine the roles of the large and small domains of Mhp-Lpl in the overall lipoylation reaction of Mhp H protein, the large and small domains were first expressed and purified (Figure 8A). Their functions were analyzed *in vitro*. As shown in Figure 8B, whether a small domain is present or absent, the large domain is able to catalyze Mhp H lipoylation. However, if the large domain was not added, then lipoate was not detected on Mhp H with the anti-lipoic acid polyclonal antibody. These results indicated that the large domain fulfills the complete catalytic function of the Mhp-Lpl enzyme, including activation of lipoic acid with ATP to form lipoyl-AMP and then catalyzing the transfer of the lipoyl moiety from lipoyl-AMP to Mhp H. However, the small domain was not required in the entire process of Mhp H lipoylation.

## Discussion

Lipoic acid is an important cofactor in most microbes. It is essential for the function of several key enzymatic complexes of central metabolism. The metabolism of lipoic acid is clearly explored in *E. coli* (Miller et al., 2000; Jordan and Cronan, 2003). Here, a new lipoate protein ligase from *M. hyopneumoniae* was verified, and its structure was solved. This analysis is the first functional identification of Lpl in *M. hyopneumoniae*. The results will help disclose the metabolism of lipoic acid in *M. hyopneumoniae* and facilitate our understanding of the process of lipoic acid metabolism in *M. hyopneumoniae*. Protein sequence alignment indicates that the Lpls are highly conserved among the different strains of *M. hyopneumoniae* (Figure S1). However, it has not been investigated how conserved lplA is in other mycoplasma species. In contrast to the high conservation of Lpls in different strains of *M. hyopneumoniae*, Lpl from *M. hyopneumoniae* has marginal identity with Lpls from other species (Figure 1B). This result is similar to that for other Lpls, such as that from *S. coelicolor* (Cao and Cronan, 2015). GcvH is an acceptor protein for activated lipoic acid in most species. Previous reports showed that *E. coli* GcvH is able to accept the lipoyl moiety transferred by lipoate protein ligase of other species (Cao and Cronan, 2015). However, our research showed that Mhp-Lpl failed to lipoylate *E. coli* GcvH. Comparison of the lipoylated sites on the *E. coli* GcvH and Mhp H showed that the lysine residues modified by lipoate attachment to *E. coli* GcvH are typical VKA sequence, while those in Mhp H are within the SKT sequence (Figure 4). This difference is probably the reason for the failure of lipoylation of *E. coli* GcvH by Mhp-Lpl. Previous studies showed that the lipoylation of lysine was not affected when the residues flanking the modified lysine were replaced with aspartate, methionine, alanine or threonine residues (Berg et al., 1994; Reche and Perham, 1999; Cao and Cronan, 2015). However, our result is not consistent with this conclusion.

In this research, the structure of Mhp-Lpl has been solved. Mhp-Lpl comprises two domains, a large N-terminal domain and a small C-terminal domain (Figure 5A). The structural arrangement of this protein is similar to that of *E. coli* (Fujiwara et al., 2005; Kim et al., 2005; McManus et al., 2006). The fully functional lipoate protein ligase has been found in many species. These ligases could be divided into three types. The first type is LplA in both *E. coli* and *S. pneumoniae*, which contains a large N-terminal domain and a small C-terminal domain. The binding pocket of the substrate resides in the interface between the two domains (Fujiwara et al., 2005, 2010). The second type is the LplA found in *S. coelicolor*, which also contains a large domain and a small domain. However, their orientation is reversed compared with that of *E. coli* (Cao and Cronan, 2015). The third type of Lpl is found in the thermophile archaeon *T. acidophilum*, in which the fully functional ligase is composed of two separate proteins, LplA and LplB, encoded by adjacent genes (Posner et al., 2009, 2013). Both LplA and LplB are required for lipoyl-AMP formation, but LplA alone is sufficient for lipoyl transferase activity (Posner et al., 2013). The crystal structure shows that the two *T. acidophilum* proteins interact to form a structure with a domain orientation similar to that of the *E. coli* LplA (Posner et al., 2013). This research indicated that Mhp-Lpl is a fully functional lipoate protein ligase. However, different from the LplA from *S. coelicolor*, in which both the large and small domains are required to activate lipoate to lipoyl-AMP and the large domain is sufficient for lipoyl transfer activity (Cao and Cronan, 2015), the large domain of Mhp-Lpl is sufficient for both the activation and transfer of lipoic acid to receptor protein (Figure 8B).

Previous studies have indicated two different pathways of lipoic acid metabolism in *E. coli* (Morris et al., 1995; Cronan et al., 2005; Cronan, 2014). These two pathways are activated in the environment with or without lipoic acid and catalyzed by different enzymes. To date, there are no reports regarding lipoic acid metabolism in *M. hyopneumoniae* and other mycoplasma species. It is unclear whether *M. hyopneumoniae* has two pathways to metabolize lipoic acid, as in *E. coli*. Here, Mhp-Lpl was identified as one enzyme responsible for lipoic acid metabolism *in vitro*. However, the environment in which these proteins play a role *in vivo*, in presence or absence of lipoic acid, remains unknown. In addition, there are two different mechanisms of lipoate synthesis described in bacteria: the *E. coli* LipB-LipA pathway and a more complex pathway in *B. subtilis* that requires two additional proteins (Christensen et al., 2011b; Martin et al., 2011). Up to now, there is no molecular system available to knock out lpl gene on the *M. hyopneumoniae* genome and therefore the function of Mhp-Lpl could not be further validated *in vivo* in this research.

Previous research indicated that the enzymes responsible for the lipoate metabolism are very important for the growth of the organisms (Wang et al., 2017; Cao et al., 2018a). Therefore, Mhp-Lpl might be a potential drug target to explore as demonstrated by the development of Lpl inhibitors which inhibited the growth of *P. falciparum* dramatically (Allary et al., 2007). This strategy is also widely applied in other metabolic enzymes. For example, the molecules which inhibit the activity of the enzymes responsible for the metabolism of biotin have been explored to inhibit the growth of *Mycobacterium tuberculosis, Staphylococcus aureus, E. coli* and so on (Brown and Beckett, 2005; Duckworth et al., 2011; Feng et al., 2016). Our work identified a functional Lpl in *M. hyopneumoniae* and the structure of this enzyme may facilitate potential drug design against *M. hyopneumoniae* infection.

## Data Availability Statement

The datasets generated for this study can be found in the Protein Data Bank (PDB), the access code 6JOM.

## Ethics Statement

The animal experiment was approved by the Institutional Animal Care and Use Committee of Harbin Veterinary Research Institute.

## Author Contributions

KZ constructed all plasmids and analyzed the enzyme activities. HC solved the protein structure. JJ and NW produced the monoclonal antibodies. GM and JH expressed the Mhp-Lpl protein. YF wrote the manuscript JX provided *M. hyopneumoniae*. HZ collected and analyzed the structural data. HL designed the experiments and wrote the manuscript.

## Conflict of Interest

The authors declare that the research was conducted in the absence of any commercial or financial relationships that could be construed as a potential conflict of interest.

## References

[B1] Adams P. D. Afonine P. V. Bunkóczi G. Bunkóczi V. B. Davis I. W. Echols N.. (2010). PHENIX: a comprehensive python-based system for macromolecular structure solution. Acta Crystallogr. D Biol. Crystallogr. 66, 213–221. 10.1107/S0907444909052925 20124702 PMC2815670

[B2] Afanador G. A. Matthews K. A. Bartee D. Gisselberg J. E. Walters M. S. Freel Meyers C. L.. (2014). Redox-dependent lipoylation of mitochondrial proteins in *Plasmodium falciparum*. Mol. Microbiol. 94, 156–171. 10.1111/mmi.12753 25116855 PMC4177315

[B3] Allary M. Lu J. Z. Zhu L. Prigge S. T. (2007). Scavenging of the cofactor lipoate is essential for the survival of the malaria parasite *Plasmodium falciparum*. Mol. Microbiol. 63, 1331–1344. 10.1111/j.1365-2958.2007.05592.x 17244193 PMC2796473

[B4] Berg A. de Kok A. Vervoort J. (1994). Sequential 1H and 15N nuclear magnetic resonance assignments and secondary structure of the N-terminal lipoyl domain of the dihydrolipoyl transacetylase component of the pyruvate dehydrogenase complex from *Azotobacter vinelandii*. Eur. J. Biochem. 221, 87–100. 10.1111/j.1432-1033.1994.tb18717.x 8068086

[B5] Brown P. H. Beckett D. (2005). Use of binding enthalpy to drive an allosteric transition. Biochemistry 44, 3112–3121. 10.1021/bi047792k 15723556

[B6] Cao X. Cronan J. E. (2015). The *Streptomyces coelicolor* lipoate-protein ligase is a circularly permuted version of the *Escherichia coli* enzyme composed of discrete interacting domains. J. Biol. Chem. 290, 7280–7290. 10.1074/jbc.M114.626879 25631049 PMC4358146

[B7] Cao X. Hong Y. Zhu L. Hu Y. Cronan J. E. (2018a). Development and retention of a primordial moonlighting pathway of protein modification in the absence of selection presents a puzzle. Proc. Natl. Acad. Sci. U.S.A. 115, 647–655. 10.1073/pnas.1718653115 29339506 PMC5789953

[B8] Cao X. Zhu L. Song X. Hu Z. Cronan J. E. (2018b). Protein moonlighting elucidates the essential human pathway catalyzing lipoic acid assembly on its cognate enzymes. Proc. Natl. Acad. Sci. U.S.A. 115, E7063–E7072. 10.1073/pnas.1805862115 29987032 PMC6064980

[B9] Chen Q. Qiu S. Li H. Lin C. Luo Y. Ren W.. (2018). A novel approach for rapid high-throughput selection of recombinant functional rat monoclonal antibodies. BMC Immunol. 19:35. 10.1186/s12865-018-0274-8 30514214 PMC6280491

[B10] Christensen Q. H. Hagar J. A. O'Riordan M. X. Cronan J. E. (2011a). A complex lipoate utilization pathway in *Listeria monocytogenes*. J. Biol. Chem. 286, 31447–31456. 10.1074/jbc.M111.273607 21768091 PMC3173067

[B11] Christensen Q. H. Martin N. Mansilla M. C. de Mendoza D. Cronan J. E. (2011b). A novel amidotransferase required for lipoic acid cofactor assembly in *Bacillus subtilis. Mol. Microbiol*. 80, 350–363. 10.1111/j.1365-2958.2011.07598.x PMC3088481 21338421

[B12] Collaborative Computational Project N (1994). The CCP4 suite: programs for protein crystallography. Acta Crystallogr. D Biol. Crystallogr. 50, 760–763. 10.1107/S0907444994003112 15299374

[B13] Cowtan K. D. Zhang K. Y. (1999). Density modification for macromolecular phase improvement. Prog. Biophys. Mol. Biol. 72, 245–270. 10.1016/s0079-6107(99)00008-5 10581970

[B14] Cronan J. E. (2014). Biotin and lipoic acid: synthesis, attachment, and regulation. EcoSal Plus 6, 1–39. 10.1128/ecosalplus.ESP-0001-2012 26442940 PMC4233344

[B15] Cronan J. E. Zhao X. Jiang Y. (2005). Function, attachment and synthesis of lipoic acid in *Escherichia coli*. Adv. Microbial Physiol. 50, 103–146. 10.1016/S0065-2911(05)50003-1 16221579

[B16] Douglas P. Kriek M. Bryant P. Roach P. L. (2006). Lipoyl synthase inserts sulfur atoms into an octanoyl substrate in a stepwise manner. Angew. Chem. Int. Ed. Engl. 45, 5197–5199. 10.1002/anie.200601910 16835858

[B17] Duckworth B. P. Geders T. W. Tiwari D. Boshoff H. I. Sibbald P. A. Barry C. E.. (2011). Bisubstrate adenylation inhibitors of biotin protein ligase from *Mycobacterium tuberculosis*. Chem. Biol. 18, 1432–1441. 10.1016/j.chembiol.2011.08.013 22118677 PMC3225891

[B18] Emsley P. Lohkamp B. Scott W. G. Cowtan K. (2010). Features and development of coot. Acta Crystallogr. D Biol. Crystallogr. 66, 486–501. 10.1107/S0907444910007493 20383002 PMC2852313

[B19] Ewald R. Hoffmann C. Florian A. Neuhaus E. Fernie A. R. Bauwe H. (2014). Lipoate-protein ligase and octanoyltransferase are essential for protein lipoylation in mitochondria of arabidopsis. Plant Physiol. 165, 978–990. 10.1104/pp.114.238311 24872381 PMC4081350

[B20] Feng J. Paparella A. S. Booker G. W. Polyak S. W. Abell A. D. (2016). Biotin protein ligase is a target for new antibacterials. Antibiotics 5:26. 10.3390/antibiotics5030026 27463729 PMC5039522

[B21] Fujiwara K. Hosaka H. Matsuda M. Okamura-Ikeda K. Motokawa Y. Suzuki M.. (2007). Crystal structure of bovine lipoyltransferase in complex with lipoyl-AMP. J. Mol. Biol. 371, 222–234. 10.1016/j.jmb.2007.05.059 17570395

[B22] Fujiwara K. Maita N. Hosaka H. Okamura-Ikeda K. Nakagawa A. Taniguchi H. (2010). Global conformational change associated with the two-step reaction catalyzed by *Escherichia coli* lipoate-protein ligase A. J. Biol. Chem. 285, 9971–9980. 10.1074/jbc.M109.078717 20089862 PMC2843243

[B23] Fujiwara K. Okamura-Ikeda K. Motokawa Y. (1992). Expression of mature bovine H-protein of the glycine cleavage system in *Escherichia coli* and *in vitro* lipoylation of the apoform. J. Biol. Chem. 267, 20011–20016. 1400316

[B24] Fujiwara K. Okamura-Ikeda K. Motokawa Y. (1997). Cloning and expression of a cDNA encoding bovine lipoyltransferase. J. Biol. Chem. 272, 31974–31978. 10.1074/jbc.272.51.31974 9405389

[B25] Fujiwara K. Toma S. Okamura-Ikeda K. Motokawa Y. Nakagawa A. Taniguchi H. (2005). Crystal structure of lipoate-protein ligase A from *Escherichia coli*. Determination of the lipoic acid-binding site. J. Biol. Chem. 280, 33645–33651. 10.1074/jbc.M505010200 16043486

[B26] Gunther S. Wallace L. Patzewitz E.-M. McMillan P. J. Storm J. Wrenger C.. (2007). Apicoplast lipoic acid protein ligase B is not essential for *Plasmodium falciparum. PLoS Pathog*. 3:e189. 10.1371/journal.ppat.0030189 18069893 PMC2134950

[B27] Hao Q. (2004). ABS: a program to determine absolute configuration and evaluate anomalous scatterer substructure. J. Appl. Crystallogr. 37, 498–499. 10.1107/S0021889804008696

[B28] Hermes F. A. Cronan J. E. (2013). The role of the *Saccharomyces cerevisiae* lipoate protein ligase homologue, Lip3, in lipoic acid synthesis. Yeast 30, 415–427. 10.1002/yea.2979 23960015 PMC3806487

[B29] Jordan S. W. Cronan J. E. Jr. (2003). The *Escherichia coli* lipB gene encodes lipoyl (octanoyl)-acyl carrier protein:protein transferase. J. Bacteriol. 185, 1582–1589. 10.1128/jb.185.5.1582-1589.2003 12591875 PMC148080

[B30] Kim D. J. Kim K. H. Lee H. H. Lee S. J. Ha J. Y. Yoon H. J.. (2005). Crystal structure of lipoate-protein ligase A bound with the activated intermediate: insights into interaction with lipoyl domains. J. Biol. Chem. 280, 38081–38089. 10.1074/jbc.M507284200 16141198

[B31] Ma Q. Zhao X. Eddine A. N. Geerlof A. Li X. Cronan J. E.. (2006). The *Mycobacterium tuberculosis* LipB enzyme functions as a cysteine/lysine dyad acyltransferase. Proc. Natl. Acad. Sci. U.S.A. 103, 8662–8667. 10.1073/pnas.0510436103 16735476 PMC1472244

[B32] Maes D. Segales J. Meyns T. Silbila M. Pieters M. Haesebrouck F. (2008). Control of *Mycoplasma hyopneumoniae* infections in pigs. Vet. Microbiol. 126, 297–309. 10.1016/j.vetmic.2007.09.008 17964089 PMC7130725

[B33] Mare C. J. Switzer W. P. (1965). New species: *Mycoplasma hyopneumoniae*; a causative agent of virus pig pneumonia. Vet. Med. Small Ani. Clin. 60, 841–846. 14323369

[B34] Martin N. Christensen Q. H. Mansilla M. C. Cronan J. E. de Mendoza D. (2011). A novel two-gene requirement for the octanoyltransfer reaction of *Bacillus subtilis* lipoic acid biosynthesis. Mol. Microbiol. 80, 335–349. 10.1111/j.1365-2958.2011.07597.x 21338420 PMC3086205

[B35] McManus E. Luisi B. F. Perham R. N. (2006). Structure of a putative lipoate protein ligase from *Thermoplasma acidophilum* and the mechanism of target selection for post-translational modification. J. Mol. Biol. 356, 625–637. 10.1016/j.jmb.2005.11.057 16384580 PMC7610907

[B36] Miller J. R. Busby R. W. Jordan S. W. Cheek J. Henshaw T. F. Ashley G. W.. (2000). *Escherichia coli* LipA is a lipoyl synthase: *in vitro* biosynthesis of lipoylated pyruvate dehydrogenase complex from octanoyl-acyl carrier protein. Biochemistry 39, 15166–15178. 10.1021/bi002060n 11106496

[B37] Morris R. J. Perrakis A. Lamzin V. S. (2003). ARP/wARP and automatic interpretation of protein electron density maps. Methods Enzymol. 374, 229–244. 10.1016/S0076-6879(03)74011-7 14696376

[B38] Morris T. W. Reed K. E. Cronan J. E. Jr. (1994). Identification of the gene encoding lipoate-protein ligase A of *Escherichia coli*. Molecular cloning and characterization of the lplA gene and gene product. J. Biol. Chem. 269, 16091–16100. 8206909

[B39] Morris T. W. Reed K. E. Cronan J. E. Jr. (1995). Lipoic acid metabolism in *Escherichia coli*: the lplA and lipB genes define redundant pathways for ligation of lipoyl groups to apoprotein. J. Bacteriol. 177, 1–10. 10.1128/JB.177.1.1-10.1995 8002607 PMC176549

[B40] Murshudov G. N. Skubák P. Lebedev A. A. Pannu N. S. Steiner R. A. Nicholls R. A.. (2011). REFMAC5 for the refinement of macromolecular crystal structures. Acta Crystallogr. D Biol. Crystallogr. 67, 355–367. 10.1107/S0907444911001314 21460454 PMC3069751

[B41] Panjikar S. Parthasarathy V. Lamzin V. S. Weiss M. S. Tucker P. A. (2005). Auto-rickshaw: an automated crystal structure determination platform as an efficient tool for the validation of an X-ray diffraction experiment. Acta Crystallogr. D Biol. Crystallogr. 61, 449–457. 10.1107/S0907444905001307 15805600

[B42] Perrakis A. Harkiolaki M. Wilson K. S. Lamzin V. S. (2001). ARP/wARP and molecular replacement. Acta Crystallogr. D Biol. Crystallogr. 57, 1445–1450. 10.1107/s0907444901014007 11567158

[B43] Pompidor G. Maury O. Vicat J. Kahn R. (2010). A dipicolinate lanthanide complex for solving protein structures using anomalous diffraction. Acta Crystallogr. D Biol. Crystallogr. 66, 762–769. 10.1107/S0907444910010954 20606256

[B44] Posner M. G. Upadhyay A. Bagby S. Hough D. W. Danson M. J. (2009). A unique lipoylation system in the Archaea. Lipoylation in Thermoplasma acidophilum requires two proteins. FEBS J. 276, 4012–4022. 10.1111/j.1742-4658.2009.07110.x 19594830

[B45] Posner M. G. Upadhyay A. Crennell S. J. Watson A. J. A. Dorus S. Danson M. J.. (2013). Post-translational modification in the archaea: structural characterization of multi-enzyme complex lipoylation. Biochem. J. 449, 415–425. 10.1042/BJ20121150 23116157

[B46] Ramaswamy A. V. Maurelli A. T. (2010). Chlamydia trachomatis serovar L2 can utilize exogenous lipoic acid through the action of the lipoic acid ligase LplA*1. J. Bacteriol*. 192, 6172–6181. 10.1128/JB.00717-10 20870766 PMC2981205

[B47] Reche P. Perham R. N. (1999). Structure and selectivity in post-translational modification: attaching the biotinyl-lysine and lipoyl-lysine swinging arms in multifunctional enzymes. EMBO J. 18, 2673–2682. 10.1093/emboj/18.10.2673 10329614 PMC1171349

[B48] Reed L. J. Hackert M. L. (1990). Structure-function relationships in dihydrolipoamide acyltransferases. J. Biol. Chem. 265, 8971–8974. 2188967

[B49] Reed L. J. Koike M. Levitch M. E. Leach F. R. (1958). Studies on the nature and reactions of protein-bound lipoic acid. J. Biol. Chem. 232, 143–158. 13549405

[B50] Schneider T. R. Sheldrick G. M. (2002). Substructure solution with SHELXD. Acta Crystallogr. D Biol. Crystallogr. 58, 1772–1779. 10.1107/s0907444902011678 12351820

[B51] Sheldrick G. M. (2002). Macromolecular phasing with SHELXE. Z Kristallogr. 217, 644–650. 10.1524/zkri.217.12.644.20662

[B52] Sheldrick G. M. (2010). Experimental phasing with SHELXC/D/E: combining chain tracing with density modification. Acta Crystallogr. D Biol. Crystallogr. 66, 479–485. 10.1107/S0907444909038360 20383001 PMC2852312

[B53] Smith A. R. Shenvi S. V. Widlansky M. Suh J. H. Hagen T. M. (2004). Lipoic acid as a potential therapy for chronic diseases associated with oxidative stress. Curr. Med. Chem. 11, 1135–1146. 10.2174/0929867043365387 15134511

[B54] Terwilliger T. C. (2003). SOLVE and RESOLVE: automated structure solution and density modification. Methods Enzymol. 374, 22–37. 10.1016/S0076-6879(03)74002-6 14696367

[B55] Wang M. Wang Q. Gao X. Su Z. (2017). Conditional knock-out of lipoic acid protein ligase 1 reveals redundancy pathway for lipoic acid metabolism in *Plasmodium berghei* malaria parasite. Parasites Vectors 10:315. 10.1186/s13071-017-2253-y 28655332 PMC5488443

[B56] Wang Q.-S. Zhang K. H. Cui Y. Wang Z.-J. Pan Q.-Y. Liu K. (2018). Upgrade of macromolecular crystallography beamline BL17U1 at SSRF. Nucl. Sci. Tech. 29:68 10.1007/s41365-018-0398-9

[B57] Zhao X. Miller J. R. Cronan J. E. (2005). The reaction of LipB, the octanoyl-[acyl carrier protein]:protein N-octanoyltransferase of lipoic acid synthesis, proceeds through an acyl-enzyme intermediate. Biochemistry 44, 16737–16746. 10.1021/bi051865y 16342964

