# Supplementary material for: Functional Identification and Structural Analysis of a New Lipoate Protein Ligase in Mycoplasma hyopneumoniae
Source: Front Cell Infect Microbiol. 2020 Apr 21;10:156. doi: 10.3389/fcimb.2020.00156 (PMC7186572; doi:10.3389/fcimb.2020.00156)
Supplement: Supplementary file 1 [file Presentation_1.pdf]

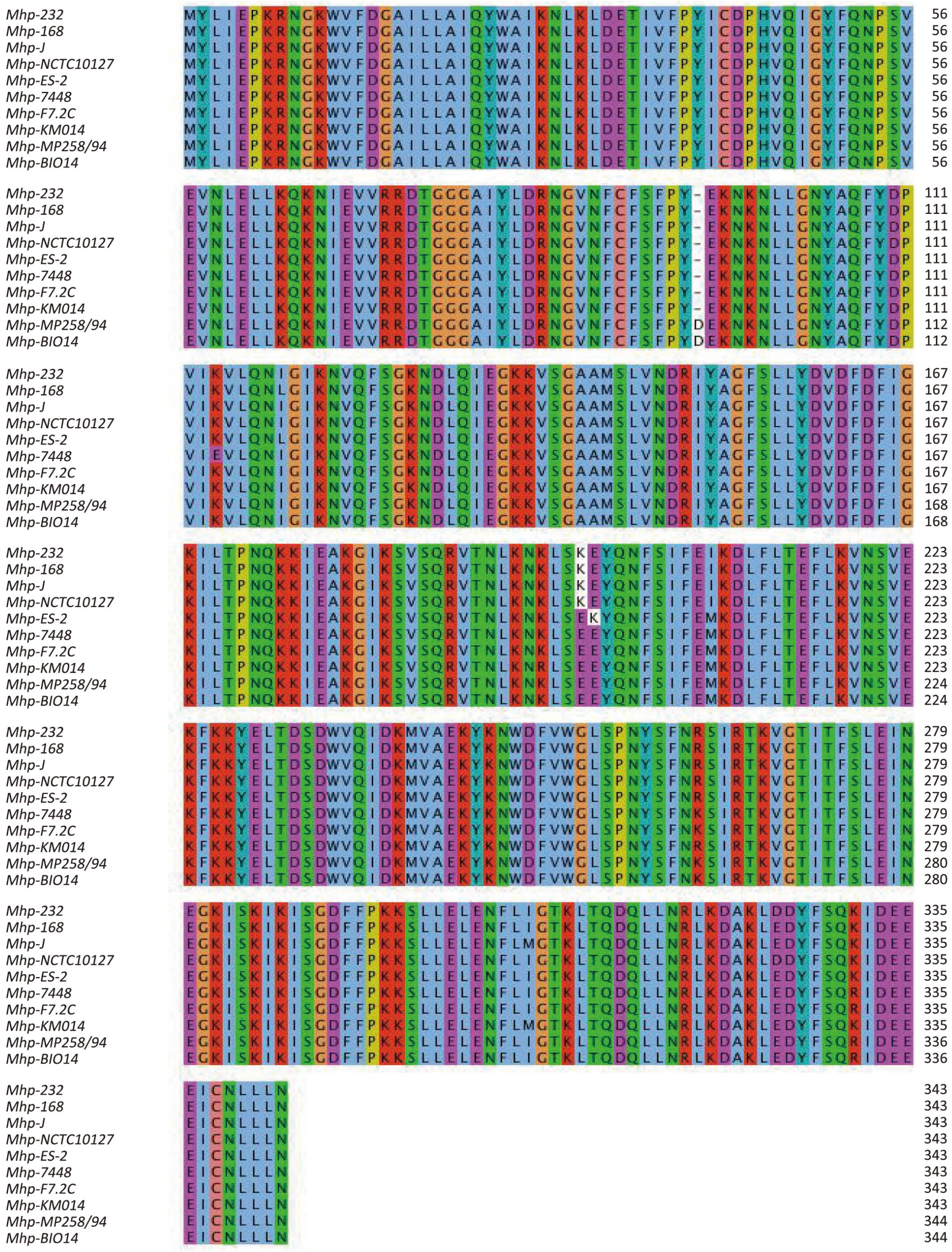

Figure S1: Protein sequences alignment among the Lpls from different strains of M. hyopneumoniae

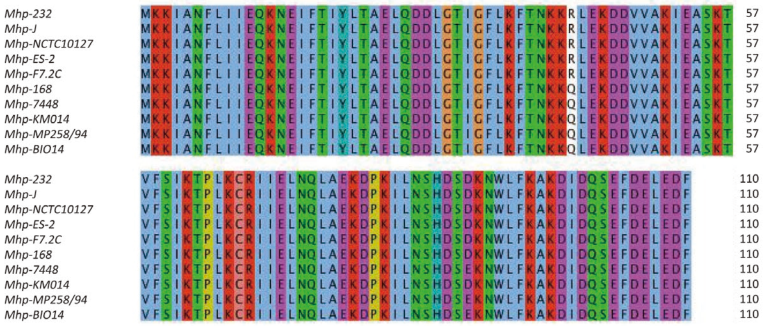

Figure S2: Protein sequences alignment among the GcvHs from different strains of *M. hyopneumoniae*
